# Prevalence of Sleep-Disordered Breathing and Its Association With Orofacial Symptoms Among Primary School Children in the Aseer Region, Saudi Arabia

**DOI:** 10.7759/cureus.45466

**Published:** 2023-09-18

**Authors:** Ayoub A Alshaikh, Reem T Alalyani, Mohammed Abdullah Aoun Alshahrani, Renad M Alshehri, Nuha S Alasmari, Shahd Abdullah A Alshahrani, Nadiyah Hussain M Almohiy, Mohammed Hassan M Asiri, Majdoleen A Abdulrahman, Abdullah Rashid S Alshahrani, Khalid S Altalhiyyah, Mahdi Muhammad M Alqahtani

**Affiliations:** 1 Family and Community Medicine, King Khalid University, Abha, SAU; 2 General Practice, King Khalid University, Abha, SAU; 3 Medicine and Surgery, King Khalid University, Abha, SAU; 4 College of Medicine, King Khalid University, Abha, SAU; 5 College of Nursing, Khamis Mushiat General Hospital, Khamis Mushiat, SAU

**Keywords:** prevalence, saudi arabia, aseer region, orofacial symptoms, sleep-disordered breathing

## Abstract

Background

Sleep-disordered breathing (SDB) is a significant health concern affecting both adults and children. However, limited research has focused on SDB and its association with orofacial symptoms in primary school children in the Aseer Region, Saudi Arabia. Understanding the prevalence and impact of SDB in this specific population is crucial for early detection and intervention. The study aims to investigate the prevalence of SDB and its associations with orofacial symptoms among primary school children.

Method

A descriptive cross-sectional survey was conducted, involving 307 primary school children aged six to 12 years in the Aseer Region. Data was collected through a web-based questionnaire, analyzing demographic information, orofacial symptoms, sleep apnea symptoms, general symptoms, growth-related symptoms, and behavioral symptoms.

Results

The study showed a balanced gender distribution, with 67.8% of children falling between ages six and nine years. Most children were Saudi nationals, and 58.6% were enrolled in primary education. Orofacial symptoms were reported by 63.5% of children, with finger-sucking and grinding teeth while sleeping being the most common. Sleep apnea symptoms affected 44.3% of children, with snoring being the prevalent symptom. General symptoms were reported by 45% of children, with daytime sleepiness being the most common. Approximately 44.6% of children exhibited sleepiness and growth-related symptoms. No statistically significant relationship was found between age and the occurrence of these symptoms.

Conclusion

The study offers valuable insights into the prevalence of SDB and its associations with orofacial symptoms among primary school children in the Aseer Region. To truly gauge the impact of interventions on SDB, further research with therapeutic interventions is warranted. In the meantime, targeted strategies and awareness initiatives are needed to address SDB in this population and enhance their overall health and quality of life.

## Introduction

Sleep-disordered breathing (SDB) encompasses a group of disorders characterized by alterations in the frequency and/or depth of breathing during sleep, ranging from simple snoring to more severe conditions like obstructive sleep apnea (OSA) [1]. This condition can affect both adults and children, with prevalence rates varying across different populations [2]. Among children, the prevalence of SDB is prominent making it a significant health concern that demands attention. However, despite its prevalence, many cases of SDB in children remain undiagnosed, leaving potential health consequences unaddressed [3]. In recent years, there has been growing recognition of the far-reaching impact of sleep-disordered breathing on a child's health and quality of life [4]. The consequences of untreated SDB can extend beyond immediate sleep disturbances and may lead to various comorbidities, including growth abnormalities, cardiovascular issues, immune disorders, and metabolic disturbances [5]. Moreover, SDB can be associated with orofacial and dentofacial characteristics, potentially linked to malocclusion and other craniofacial abnormalities, leading to further complications [6].

In the context of Saudi Arabia, the prevalence of SDB among both adults and children appears to be on the higher end of the range [7]. However, limited attention has been given to investigating SDB and its potential associations with orofacial symptoms specifically among primary school children in the Aseer Region. Understanding the prevalence and associations of SDB in this specific population is crucial for several reasons. Firstly, the primary school years represent a critical period of growth and development, and any disturbances during this phase can have long-term implications. Secondly, undiagnosed and untreated SDB during childhood may result in a cascade of health problems that persist into adulthood as reported by Simmons and Clark [8], underscoring the need for early detection and intervention. Therefore, this research aims to address this knowledge gap by investigating the prevalence of sleep-disordered breathing and its associations with orofacial symptoms among primary school children in the Aseer Region, Saudi Arabia. By shedding light on the extent of SDB in this population and its potential impact on orofacial development and overall health, the study seeks to contribute valuable insights to the field of pediatric sleep medicine and dentistry focusing on assessing the prevalence of SDB among primary school children in the Aseer Region, Saudi Arabia and investigating the associations between orofacial symptoms and sleep-disordered breathing in primary school children.

The research seeks to explore the associations between orofacial symptoms (e.g., malocclusion, mouth breathing) and the presence of SDB, providing valuable insights into potential risk factors and the interplay between orofacial development and SDB. By paving the way for better understanding and awareness, the research has the potential to lead to improved health outcomes and enhanced quality of life for children affected by SDB not only in the Aseer Region but also beyond its borders.

## Materials and methods

Study area and setting

This study was carried out in the Aseer province in the southwest region of Saudi Arabia. A web-based survey was employed to collect data efficiently, utilising the Google Forms platform. This online approach facilitates data collection from a broad spectrum of children residing within the Aseer region with the guidance and supervision of their parents or caregivers.

Survey design and sample size

In this study, a descriptive cross-sectional survey design was employed to comprehensively explore the prevalence of SDB and its associations with orofacial symptoms among primary school children. This design allowed for the collection of data from participants at a single point in time, providing valuable insights into the current state of the phenomenon under investigation. With the aim of obtaining meaningful and statistically significant results, the study targeted a total of 307 participants as its sample size. The inclusion criteria are male or female children aged six to 12 years within the Aseer region, and the exclusion criteria are children aged six to 12 years outside the Aseer region, as well as children younger than six years or older than 12 years.

Instruments

Data was meticulously collected using a structured questionnaire to gather information related to SDB and orofacial symptoms, thoughtfully developed by researchers through an extensive review of relevant literature and expert consultations. It was tailored to capture relevant information regarding snoring, breathing difficulties during sleep, orofacial symptoms, and other factors associated with sleep-disordered breathing ensuring the holistic exploration of the study's objectives. To maximize accessibility, the questionnaire was administered online through various social media platforms, utilizing Google Forms. The survey was conducted in a manner that respects the privacy and confidentiality of the participants. Informed consent was sought from each participant or their guardians prior to enrollment, emphasizing the voluntary nature of participation and the right to withdraw at any stage. To ensure adherence to ethical standards, the Research Ethical Committee of King Khalid University (KKU) granted the required ethical approval (approval number HAPO-06-B-001), underscoring the importance of protecting participants' rights and well-being throughout the study.

Data analysis

Upon completion of data collection, the gathered information was subsequently transferred to Python Jupyter Notebook version 6.4.5 (Jupyter Team, https://jupyter.org) for in-depth analysis. The collected variables underwent meticulous examination, and comparisons were conducted across various study groups. To statistically assess categorical and interval variables among these groups, the Chi-square test (χ2) was employed while continuous variables were analyzed using T-tests. A significance level of P < 0.05 was considered statistically significant, allowing for the identification of meaningful relationships and associations in the data.

## Results

Table 1 shows the demographic distribution of SDB among primary school children. The gender distribution was relatively balanced, with 50.2% (154) of the children being male and 49.8% (153) being female. The age distribution revealed that 67.8% (208) of the children fell between the ages of 6 and 9 years, while 32.2% (99) were between the ages of 10 and 12 years. Most of the children in the study, approximately 95.8% (294), were Saudi nationals, and a smaller proportion, 4.2% (13), were non-Saudi nationals. Regarding the education level, the majority of children with SDB, comprising 58.6% (180) of the sample, were enrolled in primary education. A significant portion, 14.3% (44), had not enrolled in school, possibly indicating the presence of SDB even among young children not yet attending formal education. Additionally, 8.5% (26) of the children had a medium level of education, 8.2% (25) had collegiate education, and 10.4% (32) had completed high school education.

**Table 1:** Demographic distribution of SDB among primary school children in the Aseer Region N=number of respondents; % =percentage

Variable		N	(%)
Child gender	Male	154	50.2
	Female	153	49.8
Age	From six to nine years	208	67.8
	From 10 to 12 years	99	32.2
Nationality	Saudi	294	95.8
	Non-Saudi	13	4.2
Education level	Primary	180	58.6
	He did not enroll in school	44	14.3
	Medium	26	8.5
	Collegiate	25	8.2
	High school	32	10.4

Prevalence of sleep-disordered breathing symptoms among children

Table 2 presents the prevalence of orofacial symptoms and sleep-disordered breathing in primary school children. The orofacial symptoms among the children indicated that 63.5% (195) of the children reported not suffering from any oral or facial symptoms. Among the specific symptoms observed, finger sucking, grinding teeth while sleeping, and jaw joint pain upon awakening were each reported in 7.5% (23) of the children. Other symptoms such as facial muscle pain, teeth grinding during the day, and various combinations of the symptoms were observed in smaller percentages of the children. The symptoms of sleep apnea show that 44.3% (141) of the children did not report sleep apnea symptoms. Among those with sleep apnea symptoms, snoring was the most prevalent, affecting 15.6% (48) of the children, followed by mouth breathing at 15.3% (47), and sleep apnea at 8.5% (26). Various combinations of sleep apnea symptoms were also reported but in smaller proportions. In terms of general symptoms, 45% (137) of the children did not suffer from any of the symptoms. Among those with general symptoms daytime sleepiness was the most commonly reported in 4.9% (15) of the children followed by waking up with a headache at 3.3% (10), and being overweight at 2.6% (8). Combinations of general symptoms were also reported in a significant portion of the children.

Additionally, the sleepiness and growth-related symptoms indicate that approximately 44.6% (137) of the children did not exhibit these symptoms. Among those with daytime sleepiness and growth-related symptoms, feeling lazy when waking up was the most prevalent at 12.7% (39), followed by difficulty waking up at 5.5% (17), and daytime sleepiness and teacher-noted sleepiness during the day, both reported in 4.9% (15) of the children. Combinations of these symptoms were also observed in 17.4% (54) of the children. Table 2 includes other observed symptoms. The majority, 45% (138) of the children, did not suffer from any of these additional symptoms. Among those with such symptoms, feeling in a hurry all the time was the most common at 6.8% (21), followed by attention deficit/hyperactivity at 6.2% (19), and interrupting others while speaking at 5.2% (16). Other symptoms, such as being easily distracted, not responding quickly when spoken to, being agitated, and difficulty organizing tasks, were observed in smaller percentages of the children. Combinations of these observed symptoms were reported in 20.5% (63) of the children.

**Table 2:** Prevalence of orofacial symptoms and sleep-disordered breathing in primary school children in the Aseer Region, Saudi Arabia N=number of respondents; % =percentage

	N	(%)
Does your child have any of these oral and facial symptoms		
He does not suffer from any of these symptoms	195	63.5
finger sucking	23	7.5
Grinding teeth while sleeping	23	7.5
Jaw joint pain upon awakening	19	6.2
Facial muscle pain	13	4.2
Teeth grinding during the day	10	3.3
Combinations of the symptoms	24	7.8
Does your child have any symptoms of sleep apnea		
He doesn't suffer from any of it	141	44.3
Snoring	48	15.6
mouth breathing	47	15.3
Sleep Apnea	26	8.5
snoring, breathing through the mouth	16	5.2
Sleep apnea, mouth breathing	12	3.9
Snoring, sleep apnea, mouth breathing	10	3.3
Combinations of the symptoms	7	2.3
Does your child have any of these symptoms?		
He doesn't suffer from any of it	136	44.3
He snores most of the time while sleeping	25	8.1
Snores loudly	15	4.9
difficulty breathing	14	4.6
He snores all the time while sleeping	14	4.6
Dry mouth upon awakening	13	4.2
Mouth breathing during the day	10	3.3
Wetting the bed, sleepwalking	9	2.9
He snores during the day	8	2.6
Combinations of the symptoms	80	25.1
Which of the symptoms your child has been observed (daytime sleepiness, growth)		
He does not suffer from any of these symptoms	137	44.6
lazy when he wakes up	39	12.7
It's hard to wake him up	17	5.5
Daytime sleepiness	15	4.9
The teacher notes that he is sleepy during the day	15	4.9
Wakes up with a headache	10	3.3
overweight	8	2.6
It stops growing at a normal rate	7	2.3
Lazy when he wakes up, hard to wake him up	5	1.6
Combinations of the symptoms	54	17.4
Select any of the symptoms observed in your child		
He does not suffer from any of these symptoms	138	45
He seems to be in a hurry all the time	21	6.8
Attention deficit / hyperactivity	19	6.2
Interrupts others while speaking	16	5.2
Easily distracted by external stimuli	15	4.9
Doesn't respond quickly when spoken to	13	4.2
Agitated and moves when asked to sit	13	4.2
Difficulty organizing and completing what is re	9	2.9
Combinations of the symptoms	63	20.5

Respiratory diseases among children

**Figure 1:**
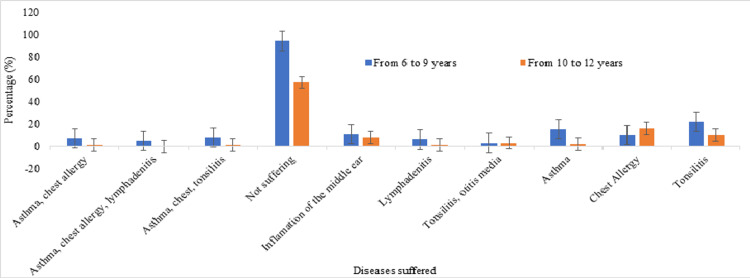
Respiratory diseases suffered among the children

Figure 1 shows the respiratory diseases suffered among the children aged six to nine years, asthma and chest allergy were reported by seven children, accounting for approximately 2.3% of this age group. Five children (1.6%) reported experiencing a combination of asthma, chest allergy, and lymphadenitis, while eight children (approximately 2.6%) had a combination of asthma, chest, and tonsillitis. A significant portion, 94 children (approximately 30.6%), did not report suffering from any of the listed respiratory diseases. Inflammation of the middle ear was reported by 11 children (approximately 3.6%), and six children (approximately 1.9%) reported having lymphadenitis. Additionally, three children (approximately 1%) experienced both tonsillitis and otitis media. Individual cases of asthma, chest allergy, and tonsillitis were reported by 15 children (approximately 4.9%), 10 children (approximately 3.3%), and 22 children (approximately 7.2%), respectively.

Among the children aged 10 to 12 years, one child (approximately 0.3%) reported having both asthma and chest allergy, while no children reported experiencing asthma, chest allergy, and lymphadenitis. A single case (approximately 0.3%) of asthma, chest, and tonsillitis was reported. The majority of children in this age group, 57 children (approximately 18.6%), did not suffer from any of the listed respiratory diseases. Other reported conditions include inflammation of the middle ear, lymphadenitis, and both tonsillitis and otitis media, with 8 children (approximately 2.6%), 1 child (approximately 0.3%), and 3 children (approximately 1%) experiencing these respective conditions. Individual cases of asthma, chest allergy, and tonsillitis were reported by two children (approximately 0.7%), 16 children (approximately 5.2%), and 10 children (approximately 3.3%), respectively.

Association of sleep-disordered breathing symptoms among children across different age groups

Table 3 assesses the relationship between children's age and the various orofacial symptoms among primary school children. Regarding oral and facial symptoms, the data shows that the majority of children in both age groups reported not suffering from any of the listed symptoms. The most prevalent symptoms in the younger group (from six to nine years) were finger sucking (5.5%) and grinding teeth while sleeping (5.9%). In the older group (from 10 to 12 years), chest allergy (5.2%) and snoring (4.6%) were the most frequently reported symptoms. However, the P-value for this set of symptoms is 0.603, indicating that there is no statistically significant relationship between age and the occurrence of these symptoms. Moving on to symptoms of sleep apnea, a similar pattern emerges. The majority of children in both age groups do not suffer from any sleep apnea symptoms. Snoring was the most prevalent symptom in both groups (11.1% in the younger group and 4.6% in the older group). However, once again, the P-value for this set of symptoms is 0.642, suggesting that there is no statistically significant relationship between age and the occurrence of sleep apnea symptoms. Similarly, for other general symptoms such as daytime sleepiness and growth-related symptoms, there is no statistically significant relationship with age (P-value = 0.761).

**Table 3:** Assessing the relationship between the children's age and the various orofacial symptoms among primary school children in the Aseer Region, Saudi Arabia

	Childs age				
	From six to nine years	(%)	From 10 to 12 years	(%)	P Value
Does your child have any of these oral and facial symptoms					0.603
He does not suffer from any of these symptoms	0	0.0	1	0.3	
finger sucking	17	5.5	6	2.0	
Grinding teeth while sleeping	18	5.9	5	1.6	
Jaw joint pain upon awakening	2	0.7	1	0.3	
Facial muscle pain	8	2.6	5	1.6	
Teeth grinding during the day	8	2.6	2	0.7	
Combinations of the symptoms	164	53.4	70	22.8	
Does your child have any symptoms of sleep apnea					
He doesn't suffer from any of it	96	31.3	45	14.7	0.778
snoring	34	11.1	14	4.6	
mouth breathing	29	9.4	18	5.9	
Sleep Apnea	19	6.2	7	2.3	
snoring, breathing through the mouth	10	3.3	6	2.0	
Sleep apnea, mouth breathing	10	3.3	2	0.7	
Snoring, sleep apnea, mouth breathing	5	1.6	5	1.6	
Combinations of the symptoms	5	1.6	2	0.7	
Does your child have any of these symptoms?					
He doesn't suffer from any of it	94	30.6	42	13.7	0.642
He snores most of the time while sleeping	12	3.9	9	2.9	
Snores loudly	18	5.9	7	2.3	
difficulty breathing	10	3.3	4	1.3	
Dry mouth upon awakening	13	4.2	6	2.0	
Mouth breathing during the day	6	2.0	0	0.0	
Wetting the bed, sleepwalking	7	2.3	2	0.7	
He snores during the day	17	5.5	7	2.3	
Combinations of the symptoms	37	12.1	16	5.2	
Which of the symptoms your child has been observed (daytime sleepiness, growth)					0.761
He does not suffer from any of these symptoms	96	31.3	41	13.4	
lazy when he wakes up	24	7.8	15	4.9	
It's hard to wake him up	3	1	1	0.3	
Daytime sleepiness	12	3.9	3	1	
The teacher notes that he is sleepy during the day	11	3.6	4	1.3	
Wakes up with a headache	5	1.6	5	1.6	
overweight	4	1.3	4	1.3	
It stops growing at a normal rate	4	1.3	2	0.7	
Lazy when he wakes up, hard to wake him up	51	16.6	22	7.2	
Combinations of the symptoms					
Select any of the symptoms observed in your child					
He does not suffer from any of these symptoms	3	1.0	5	1.6	
He seems to be in a hurry all the time	3	1.0	0	0.0	
Attention deficit / hyperactivity	11	3.6	8	2.6	0.941
Interrupts others while speaking	10	3.3	6	2.0	
Easily distracted by external stimuli	2	0.7	3	1.0	
Doesn't respond quickly when spoken to	3	1.0	0	0.0	
Agitated and moves when asked to sit	10	3.3	3	1.0	
Difficulty organizing and completing what is required	6	2.0	4	1.3	
Combinations of the symptoms	138	45.0	92	30.0	

Finally, the data on behavioral symptoms observed in the children shows no statistically significant relationship with age (P value = 0.941). Most children in both age groups did not suffer from any of these symptoms. The most commonly observed symptom in both groups was being in a hurry all the time, with 1.0% of children in the younger group and none in the older group. Overall, the data indicates that there is no statistically significant relationship between the child's age and the various orofacial, sleep apnea, general, and behavioral symptoms among primary school children in the Aseer Region. The majority of children in both age groups did not report suffering from these symptoms, and the prevalence of specific symptoms does not significantly vary with age.

## Discussion

The research results provide valuable insights into the demographic distribution and prevalence of sleep-disordered breathing (SDB) symptoms among primary school children in the Aseer Region, Saudi Arabia. SDB in children differs from adults in a number of ways, including presenting symptoms and treatment [9]. The relatively balanced gender distribution of the sample indicates that SDB does not show a significant gender bias, affecting both male and female children almost equally. The age distribution is notable, with a higher proportion of children (67.8%) falling within the age range of six to nine years. This finding suggests that SDB is more prevalent among younger children in the primary school age group. This result agrees with Boss et al. [10] study, which reported the prevalence of SBD among elementary school children. The lower proportion of children (32.2%) in the 10 to 12 years age range could indicate that some children may outgrow SDB symptoms as they get older, a similar study by Löfstrand-Tideström and Hultcrantz [11] confirms that children who snore at the age of four years seldom “grow out of it” by the age of six years. However, further longitudinal research is needed to confirm this hypothesis. Regarding education levels, the highest percentage of children with SDB are enrolled in primary education (58.6%). This finding suggests that SDB may affect children during their early educational years, potentially impacting their academic performance and overall well-being. The finding by Galland et al. [12] affirms that SDB was significantly associated with poorer academic performance for core academic domains related to language arts. The significant percentage of children (14.3%) who had not yet enrolled in school but still showed SDB symptoms raises concerns about the early onset and detection of this condition even before formal education begins. This argument aligns with Byars et al. [13] whose finding reported that parent-reported sleep problems during infancy and early toddlerhood (6-24) months.

Additionally, the distribution of orofacial symptoms among the children highlights that a considerable portion (63.5%) of the children did not suffer from any oral or facial symptoms. The presence of finger sucking, grinding teeth while sleeping, and jaw joint pain upon awakening in a minority of children may indicate potential risk factors or habits contributing to SDB development. Children with respiratory conditions or orofacial symptoms were at higher risk of sleep-disordered breathing Baidas et al. [14]. The prevalence of sleep apnea symptoms is noteworthy, with a substantial percentage of children (44.3%) not reporting any of these symptoms. However, among those with sleep apnea symptoms, snoring appears to be the most common issue (15.6%), a result which agrees with Romero et al. [15] that snoring is a prevalent symptom of obstructive sleep apnea. Sleep apnea, mouth breathing, and combinations of these symptoms are also observed, albeit in smaller proportions. These findings underscore the importance of early detection and intervention for sleep apnea among primary school children to ensure their overall health and well-being. Furthermore, the presence of general symptoms, daytime sleepiness, growth-related symptoms, and other behavioral symptoms warrants attention. A considerable proportion of children did not exhibit these symptoms, indicating that not all children with SDB may experience these associated issues. However, identifying symptoms like feeling lazy when waking up, daytime sleepiness, and being in a hurry all the time in some children highlights the potential impact of SDB on their daily lives and overall health. The research results provide a comprehensive picture of the prevalence and impact of SDB among primary school children in the Aseer Region. These findings can serve as a foundation for further research and targeted interventions to address SDB in this population. Early detection and appropriate management of SDB can potentially lead to improved health outcomes and overall well-being for affected children, ultimately contributing to better academic performance and quality of life.

However, the study's cross-sectional design restricts our ability to establish causal relationships between variables. While the findings highlight prevalent patterns, they do not provide a comprehensive understanding of the temporal evolution of SDB symptoms. Secondly, the data collection method employed, which relies on a web-based survey using Google Forms, might introduce selection bias, as it excludes individuals without internet access or those not familiar with digital tools. This could potentially affect the representation of certain segments of the population, leading to skewed prevalence estimates. Additionally, the study's reliance on self-reported symptoms introduces the possibility of recall bias, as children or their parents might not accurately remember or report certain symptoms. Furthermore, the lack of detailed medical history and clinical assessments limits the study's ability to comprehensively account for potentially confounding factors. The study's focus on a specific region, the Aseer Province, may also limit the generalizability of the findings to broader populations or different geographic locations. To address this limitation in future studies, we intend to implement more comprehensive data collection methods. Specifically, we plan to incorporate detailed medical history questionnaires and conduct comprehensive clinical assessments for participants. This approach will allow us to account for potential confounding variables more effectively and enhance the internal validity of our research; also we plan to expand the scope of our research by including multiple regions or areas within the Aseer Province in future studies.

## Conclusions

The research findings provide valuable insights into sleep-disordered breathing (SDB) among primary school children in the Aseer Region, Saudi Arabia. The study reveals a relatively balanced gender distribution, with a higher prevalence of SDB symptoms in children aged six to nine years. Most affected children are Saudi nationals, and early detection during primary education is crucial. Orofacial symptoms and sleep apnea symptoms highlight the need for timely intervention. Overall, the results call for targeted strategies to improve the well-being of affected children in the region.
